# Social Determinants and Prevention Strategies in the HIV Epidemic: The National Center for HIV/AIDS, Viral Hepatitis, STD, and TB Prevention (NCHHSTP) Database Analysis

**DOI:** 10.7759/cureus.84456

**Published:** 2025-05-20

**Authors:** Edediong Ekarika, Charity Iheagwara, Adaora T Amadi, Patra C Ezeamii, Michael O Oluwalana, Linda C Ihuoma, Ogechukwu H Nnabude, Nenrot S Gopep, Okelue E Okobi, Nnenna B Emejuru

**Affiliations:** 1 Public Health, Emory University, Rollins School of Public Health, Atlanta, USA; 2 Medicine, All Saints University School of Medicine, Roseau, DMA; 3 Infectious Diseases, Saint Michael's Medical Center, Newark, USA; 4 Family Medicine, Enugu State University, College of Medicine, Enugu, NGA; 5 Epidemiology and Public Health, Jiann-Ping Hsu College of Public Health, Georgia Southern University, Statesboro, USA; 6 Faculty of Medicine, Memorial University of Newfoundland, St. John's, CAN; 7 Internal Medicine, University of Port Harcourt Teaching Hospital, Choba, NGA; 8 Dermatology, Psychiatry, Cardiology, Internal Medicine, Family Medicine, Windsor University School of Medicine, St. Kitts, KNA; 9 Community Medicine, Federal Medical Center, Keffi, NGA; 10 Public Health, Georgia Southern University, Statesboro, USA; 11 Family Medicine, IMG Research Academy &amp; Consulting, Miami, USA; 12 Family Medicine, Larkin Community Hospital Palm Springs Campus, Hialeah, USA; 13 Family Medicine, Lakeside Medical Center, Belle Glade, USA; 14 Psychiatry and Behavioral Sciences, College of Medicine, Imo State University, Orlu, NGA

**Keywords:** hiv, hiv diseases, hiv epidemic, hiv prevention, social determinant

## Abstract

Background: The human immunodeficiency virus (HIV) epidemic remains a significant public health challenge, with social determinants and prevention strategies playing a crucial role in disease outcomes. While advancements in treatment and prevention have led to improvements in viral suppression and healthcare access, disparities in healthcare remain, particularly among vulnerable populations.

Aim: This study analyzes HIV epidemiological trends, healthcare access, and social determinants influencing the HIV epidemic in the United States from 2018 to 2022, using data from the National Center for HIV/AIDS, Viral Hepatitis, STD, and TB Prevention (NCHHSTP) database.

Method: A retrospective analysis of national surveillance data was conducted to assess trends in HIV-related mortality, viral suppression, incidence, prevalence, and healthcare access. Key indicators such as knowledge of HIV status, PrEP coverage, linkage to care, HIV stigma, and unstable housing were evaluated. Data were analyzed for temporal trends, with a focus on the impact of the COVID-19 pandemic on HIV outcomes.

Results: The findings indicate a reduction in new HIV infections and an increase in HIV prevalence, suggesting improvements in diagnosis and treatment. Although AIDS-related and HIV-related deaths spiked during the COVID-19 pandemic, viral suppression rates steadily improved. Healthcare access remained stable, with increased PrEP coverage and linkage to care. However, persistent barriers such as HIV stigma and unstable housing continued to affect health outcomes.

Conclusion: The study highlights significant progress in HIV prevention and care but underscores the need for targeted interventions addressing social determinants. Continued investment in equitable healthcare access, stigma reduction, and housing stability is essential for sustaining control of the HIV epidemic.

## Introduction

The HIV epidemic remains a significant global public health challenge, with its impact disproportionately affecting specific populations due to a complex interplay of biological, social, and structural determinants [1]. Despite substantial advancements in antiretroviral therapy (ART) and preventive strategies, disparities in transmission rates, diagnosis, and treatment accessibility persist [2]. Socioeconomic status, education, healthcare access, and stigma continue to shape the epidemic's trajectory, underscoring the need for a comprehensive approach to intervention [3]. Utilizing large-scale epidemiological data, researchers can gain deeper insights into these determinants, enabling the development of targeted, evidence-based prevention and treatment strategies [4].

HIV epidemiology has evolved, disproportionately affecting marginalized groups. Despite ART advancements, new infections persist. Surveillance data from institutions like the National Center for HIV/AIDS, Viral Hepatitis, STD, and TB Prevention (NCHHSTP) help track trends, risk factors, and intervention effectiveness, guiding public health strategies [5]. At the end of 2023, an estimated 39.9 million people were living with HIV globally, including 38.6 million adults and 1.4 million children, which underscores the continuing worldwide burden of disease. In 2022, of the 39 million people living with HIV globally, 53% were women and girls. HIV-related mortality remains significant, highlighting the ongoing global burden of the disease [4].

HIV is a retrovirus primarily targeting CD4+ T cells, but virological mechanisms (e.g., reverse transcription, mutation) are beyond the scope of this study [1]. The virus gains entry through specific surface receptors, undergoes reverse transcription, and integrates its genetic material into the host genome, enabling persistent replication [6]. This gradual depletion of CD4+ T cells weakens immune defenses, increasing vulnerability to opportunistic infections and AIDS-related complications [1]. While antiretroviral therapy (ART) effectively suppresses viral replication and delays disease progression, challenges such as treatment adherence, drug resistance, and late-stage diagnoses continue to hinder optimal outcomes [7]. Additionally, emerging mutations in the viral genome can compromise therapeutic efficacy, necessitating continuous advancements in drug development and monitoring strategies [8]. A deeper understanding of HIV pathophysiology is crucial for enhancing treatment regimens, improving long-term patient survival, and informing the development of novel therapeutic interventions aimed at achieving sustained viral suppression and functional cure strategies [1,8-10].

The NCHHSTP database serves as a valuable resource for epidemiological research, offering comprehensive surveillance data on HIV and other infectious diseases [9,10]. This database compiles information from national reporting systems, healthcare facilities, and community-based studies, providing a robust framework for analyzing disease patterns, risk factors, and intervention effectiveness. By utilizing this dataset, researchers can assess trends in HIV incidence, prevalence, and disparities across different populations. Additionally, the database facilitates the evaluation of public health programs, helping policymakers tailor interventions to address high-risk groups and emerging challenges in HIV prevention and treatment.

This study aims to analyze the roles of HIV stigma and unstable housing in shaping the HIV epidemic and assess the effectiveness of current prevention strategies using the NCHHSTP database. By examining demographic disparities, socioeconomic influences, and healthcare accessibility, the study seeks to identify key factors contributing to HIV transmission and treatment gaps. The findings will provide insights into potential policy changes and targeted interventions to mitigate disease burden and improve health equity. Through data-driven approaches, this research aims to enhance public health strategies for HIV prevention, ultimately contributing to the broader goal of reducing new infections and improving outcomes for affected populations.

## Materials and methods

Data source and study design

This study utilized data from the NCHHSTP database, a comprehensive repository of surveillance and epidemiological data on infectious diseases in the United States. The study followed a cross-sectional design, analyzing retrospective data from reported HIV cases over a specified period. The dataset included demographic, clinical, and behavioral information collected through national surveillance systems, enabling an in-depth examination of the role of social determinants in HIV transmission and prevention efforts.

Study participants and questionnaires

The study population comprised all individuals with a confirmed HIV diagnosis reported to NCHHSTP during the study period. Because reporting of HIV diagnoses to NCHHSTP is required by law in all 50 states and U.S. territories, our sample constitutes a near‑complete enumeration of diagnosed cases, supporting generalizability to the U.S. population of people living with HIV. Inclusion criteria included a confirmed HIV diagnosis date within the study window, complete documentation of age, sex, race/ethnicity, and state of residence, and availability of data on HIV stigma and housing status. Records were excluded if key variables (e.g., diagnosis date, stigma score, housing status) were entirely missing. The database contained self-reported information from structured questionnaires used in surveillance programs, capturing data on risk behaviors, healthcare access, socioeconomic status, and preventive measures such as pre-exposure prophylaxis (PrEP) usage and HIV testing frequency.

Data collection and quality assurance

Data in the NCHHSTP database were collected through national reporting systems, healthcare facilities, and public health surveillance programs. Standardized protocols ensured data reliability and validity, including double-entry verification and routine cross-checking with healthcare records. Quality assurance measures involved automated data validation, periodic audits, and statistical checks to identify inconsistencies or missing values. Additionally, duplicate records were removed, and imputations were applied where appropriate to minimize bias. The dataset underwent periodic updates to reflect the most recent trends in HIV epidemiology and prevention efforts.

Variables of interest

We analyzed three primary domains of variables to understand HIV-related outcomes comprehensively: (1) HIV Outcomes & Mortality, which included measures such as AIDS-related deaths, overall HIV-related mortality, and viral suppression rates; (2) HIV Burden & Epidemiology, encompassing variables like AIDS classifications, estimated incidence, prevalence of diagnosed and undiagnosed HIV, and new diagnoses; and (3) Healthcare Access & Social Factors, covering standardized HIV stigma scores, knowledge of HIV status, linkage to care, PrEP coverage, receipt of medical care, and unstable housing. For variables with less than 10% of missing data, we applied multiple imputation using chained equations, assuming data were missing at random. Variables with more than 10% missingness were assessed for potential bias, and if imputation was not deemed reliable, we used listwise deletion in relevant analyses, supplemented by sensitivity analyses comparing findings from the complete and imputed datasets.

Data analysis and statistical methods

Descriptive statistics, including frequencies and percentages, were used to summarize demographic characteristics and key variables. Bivariate analyses, such as chi-square tests and ANOVA, were conducted to examine associations between social determinants and HIV-related outcomes. A significant level of p < 0.05 was set for all statistical tests. Data analysis was conducted using IBM Corp. Released 2024. IBM SPSS Statistics for Windows, Version 30. Armonk, NY: IBM Corp, ensuring robust analytical methodologies.

Ethical considerations

As this study utilized de-identified secondary data from a publicly available database, it did not require direct patient consent. Because the NCHHSTP data are de‑identified and collected under mandatory public health surveillance statutes, this project was classified as non‑human subjects research and exempt from IRB review per 45 CFR 46.101(b)(4). Data confidentiality was maintained through encrypted storage on secure Centers for Disease Control and Prevention (CDC) servers in compliance with the Health Insurance Portability and Accountability Act (HIPAA) and CDC data protection policies.

## Results

**Figure 1:**
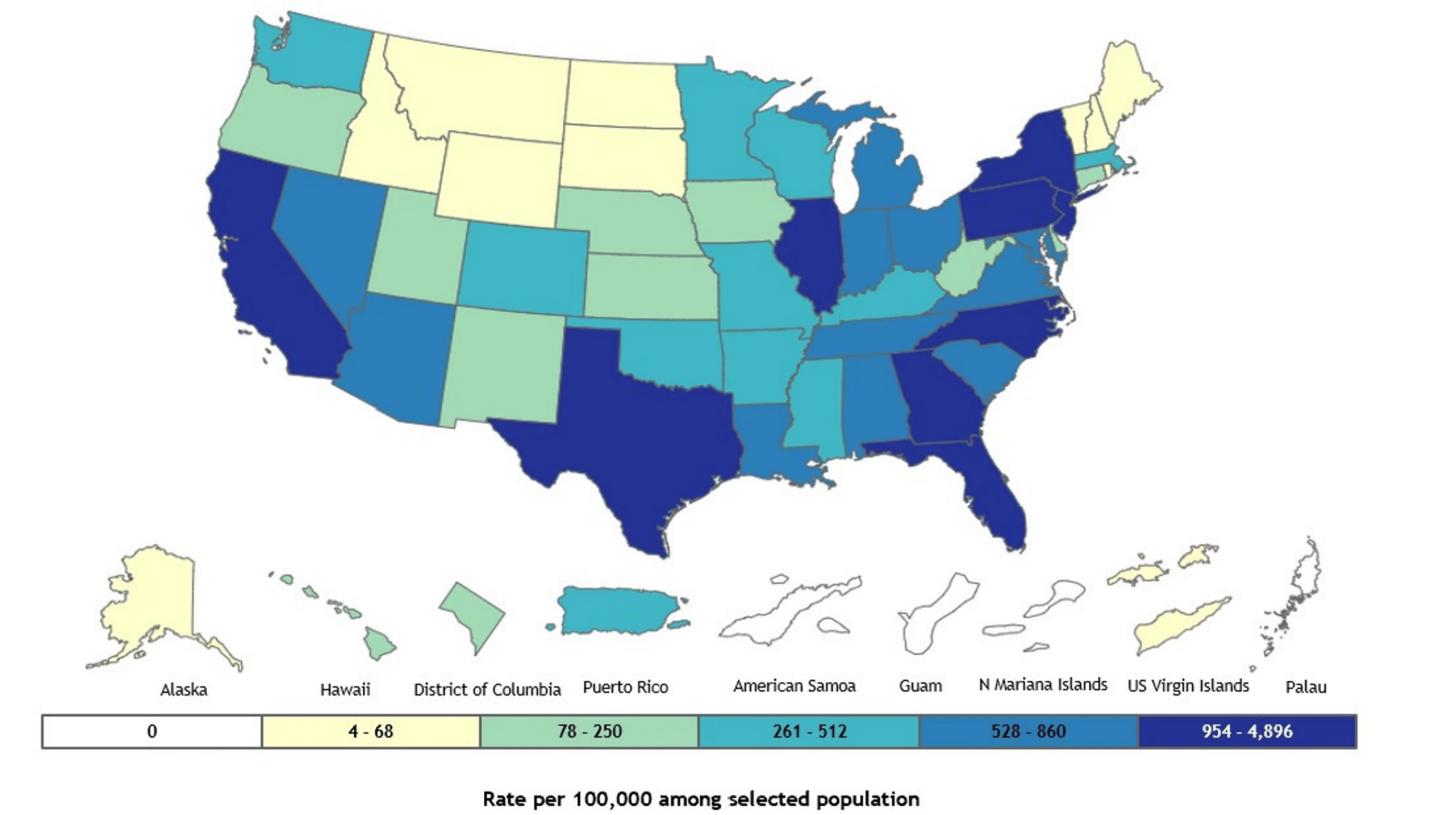
HIV diagnoses in the United States HIV: Human Immunodeficiency Virus Data source: UNAIDS data 2021. https://gis.cdc.gov/grasp/nchhstpatlas/maps.html [10].

The results comprehensively overview HIV epidemiological trends, healthcare access, and social determinants from 2018 to 2022. By analyzing key indicators such as mortality rates, viral suppression, and healthcare coverage, this study identifies significant progress and persistent challenges in the HIV epidemic. The findings highlight the impact of the COVID-19 pandemic on HIV outcomes while emphasizing the ongoing need for targeted interventions to address disparities in care and prevention. Figure 1 presents state-level data on HIV diagnoses in 2022 for individuals aged 13 years and older across all races, ethnicities, sexes, and transmission categories.

Based on HIV outcomes and mortality

Between 2018 and 2022, the number of HIV/AIDS-related deaths fluctuated, with a notable peak in 2021 (15,116 cases, rate: 5.4 per 100,000) and a dip in 2019 (12,471 cases, rate: 4.5 per 100,000). The highest reported mortality occurred during the COVID-19 pandemic in 2020, with 14,529 cases (rate: 5.2 per 100,000). While a decrease was observed in 2022 (14,208 cases, rate: 5 per 100,000), the numbers remained elevated compared to 2018 and 2019. Table 1 illustrates HIV-related mortality, including AIDS-related deaths and overall HIV deaths, highlighting the impact of the disease on affected populations.

**Table 1:** Trends in AIDS deaths, HIV deaths, and HIV viral suppression AIDS: Acquired Immunodeficiency Syndrome, CI: Confidence Interval, HIV: Human Immunodeficiency Virus, RSE: Relative Standard Error. P-value and F-value were computed using one-way ANOVA.

Year	AIDS deaths		HIV deaths		HIV viral suppression	
	Cases (95% CI RSE)	Rate per 100000 (95% CI)	Cases (95% CI RSE)	Rate per 100000 (95% CI)	Cases (95% CI RSE)	Percent (95% CI RSE)
2018	12,649	4.6	16,192	5.9	565,195	64.7
2019	12,471	4.5	16,266	5.9	605,756	65.5
2020	14,529	5.2	19,136	6.8	609,654	64.6
2021	15,116	5.4	20,181	7.2	635,300	65.9
2022	14,208	5	18,937	6.7	663,121	65.1
P-value	P<0.05		P<0.05		P<0.05	
F-value	7667044.569		7150233.245		6828805.264	

HIV-related deaths followed a similar increasing trend, with the highest number recorded in 2021 (20,181 cases, rate: 7.2 per 100,000). The lowest figures were reported in 2019 (16,266 cases, rate: 5.9 per 100,000). The COVID-19 pandemic in 2020 was associated with an increase in HIV-related deaths (19,136 cases, rate: 6.8 per 100,000), which persisted into 2022 (18,937 cases, rate: 6.7 per 100,000), though slightly lower than in 2020 and 2021.

The percentage of individuals achieving HIV viral suppression showed a general increasing trend over the years. The lowest reported percentage was in 2018 (64.7%, 565,195 cases), while the highest occurred in 2021 (65.9%, 635,300 cases). A slight decline was observed in 2022 (65.1%, 663,121 cases), although the absolute number of individuals with viral suppression increased over time. Notably, during the COVID-19 pandemic in 2020, viral suppression was recorded at 64.6% (609,654 cases), indicating potential disruptions in healthcare services. The p-value for AIDS deaths, HIV deaths, and HIV viral suppression is p < 0.05, indicating a statistically significant difference over the years.

Based on HIV burden and epidemiology

The number of AIDS classifications fluctuated between 2018 and 2022 (Table 2). The highest number was reported in 2018 (17,106 cases, rate: 6.2 per 100,000), followed by a gradual decrease through 2020 (14,353 cases, rate: 5.1 per 100,000) before increasing again in 2022 (16,687 cases, rate: 5.9 per 100,000). AIDS prevalence remained relatively stable over the years, with slight variations, reaching 532,646 cases in 2022 (rate: 188.6 per 100,000).

**Table 2:** Trends in AIDS classifications, prevalence, HIV incidence, and HIV prevalence in the U.S. AIDS: Acquired Immunodeficiency Syndrome, CI: Confidence Interval, HIV: Human Immunodeficiency Virus, RSE: Relative Standard Error. P-value and F-value were computed using one-way ANOVA.

Year	AIDS classifications		AIDS prevalence		Estimated HIV incidence		Estimated HIV prevalence (undiagnosed and diagnosed)		HIV diagnoses		HIV prevalence	
	Cases (95% CI RSE)	Rate per 100000 (95% CI)	Cases (95% CI RSE)	Rate per 100000 (95% CI)	Cases (95% CI RSE)	Rate per 100000 (95% CI)	Cases (95% CI RSE)	Rate per 100000 (95% CI)	Cases (95% CI RSE)	Rate per 100000 (95% CI)	Cases (95% CI RSE)	Rate per 100000 (95% CI)
2018	17,106	6.2	525,214	191.5	36,200 (34,600 - 37,700, 2.2)	13.2 (12.6 - 13.7)	1,167,500 (1,160,500 - 1,174,600, 0.3)	425.7 (423.1 - 428.2)	37,132	13.5	1,014,481	369.9
2019	16,410	5.9	529,196	191.7	35,100 (33,400 - 36,800, 2.5)	12.7 (12.1 - 13.3)	1,188,300 (1,181,000 - 1,195,600, 0.3)	430.5 (427.8 - 433.1)	36,350	13.2	1,036,801	375.6
2020	14,353	5.1	529,066	189.2	34,200 (32,100 - 36,300, 3.1)	12.2 (11.5 - 13.0)	1,199,700 (1,192,100 - 1,207,300, 0.3)	429.1 (426.4 - 431.8)	30,317	10.8	1,049,709	375.4
2021	15,992	5.7	530,044	188.7	32,700 (30,400 - 34,900, 3.5)	11.6 (10.8 - 12.4)	1,215,900 (1,208,000 - 1,223,900, 0.3)	432.9 (430.1 - 435.7)	35,671	12.7	1,068,119	380.3
2022	16,687	5.9	532,646	188.6	31,800 (29,200 - 34,400, 4.1)	11.3 (10.3 - 12.2)	1,238,000 (1,229,600 - 1,246,400, 0.3)	438.2 (435.3 - 441.2)	37,601	13.3	1,092,023	386.6
P-value	P<0.05		P<0.05		P<0.05		P<0.05		P<0.05		P<0.05	
F-value	7603378.519		3450566.251		6491563.349		520375.8944		5422951.938		324208.489	

Estimated HIV incidence declined from 36,200 cases in 2018 (rate: 13.2 per 100,000) to 31,800 cases in 2022 (rate: 11.3 per 100,000). This downward trend suggests a reduction in new HIV infections over time. Estimated HIV prevalence, which includes both diagnosed and undiagnosed cases, showed a consistent increase, reaching 1,238,000 cases in 2022 (rate: 438.2 per 100,000), up from 1,167,500 cases in 2018 (rate: 425.7 per 100,000). This trend highlights improvements in HIV identification and care services.

HIV diagnoses fluctuated over the years, peaking in 2022 with 37,601 cases (rate: 13.3 per 100,000). The lowest number was recorded during the COVID-19 pandemic in 2020 (30,317 cases, rate: 10.8 per 100,000), suggesting potential disruptions in testing and diagnosis services. HIV prevalence also followed an increasing trend, rising from 1,014,481 cases in 2018 (rate: 369.9 per 100,000) to 1,092,023 cases in 2022 (rate: 386.6 per 100,000), reflecting both improved access to care and a growing population of individuals living with HIV. The p-value for all HIV burden and epidemiology variables indicated statistically significant (p < 0.05) differences over the years.

Based on healthcare access and social factors

HIV stigma, measured by the percentage of individuals reporting no suppression, showed a general decline over time (Table 3). The highest rate was observed in 2018 (31.2%), which gradually decreased to 28.4% in 2020 during the COVID-19 pandemic. A slight increase was noted in 2022 (29.3%), suggesting potential challenges in reducing stigma despite improvements in HIV care. The percentage of individuals aware of their HIV status increased steadily from 85.9% in 2018 to 87.2% in 2022. The lowest recorded percentage was in 2018 (1,003,086 cases, 85.9%), while the highest was in 2022 (1,079,751 cases, 87.2%). Notably, during the COVID-19 pandemic in 2020, knowledge of status remained relatively stable at 86.5%, indicating resilience in diagnostic efforts despite healthcare disruptions.

**Table 3:** Trends in healthcare access and social factors in the U.S. CI: Confidence Interval, HIV: Human Immunodeficiency Virus, PrEP: Pre-Exposure Prophylaxis, CI: Confidence interval, RSE: Relative Standard Error. P-value and F-value were computed using one-way ANOVA.

Year	HIV Stigma		Knowledge of Status		Linkage to HIV care		PrEP coverage and number of persons prescribed		Receipt of HIV medical care		Unstable Housing or Homelessness	
	Cases (95% CI RSE)	Rate per 100000 (95% CI)	Cases (95% CI RSE)	Percent (95% CI RSE)	Cases (95% CI RSE)	Percent (95% CI RSE)	Cases (95% CI RSE)	Percent (95% CI RSE)	Cases (95% CI RSE)	Percent (95% CI RSE)	Cases (95% CI RSE)	Rate per 100000 (95% CI)
2018	No Suppression	31.2 (30.3 - 32.1)	1,003,086	85.9 (85.4 - 86.4, 0.3)	26,858	80.2	221,026	18.2	661,816	75.7	No Suppression	21.0 (19.5 - 22.6)
2019	No Suppression	30.7 (29.2 - 32.1)	1,025,126	86.3 (85.7 - 86.8, 0.3)	27,479	81.3	275,794	22.7	703,023	76	No Suppression	19.8 (18.0 - 21.6)
2020	No Suppression	28.4 (27.7 - 29.2)	1,037,822	86.5 (86.0 - 87.1, 0.3)	23,419	82.4	301,324	24.8	699,587	74.1	No Suppression	17.2 (15.2 - 19.2)
2021	No Suppression	28.8 (27.6 - 30.1)	1,056,027	86.8 (86.3 - 87.4, 0.3)	27,535	81.9	366,359	30.1	726,340	75.3	No Suppression	17.0 (15.3 - 18.6)
2022	No Suppression	29.3 (27.9 - 30.7)	1,079,751	87.2 (86.6 - 87.8, 0.3)	29,753	81.6	437,425	36	769,575	75.6	No Suppression	17.9 (16.5 - 19.3)
P-value	P<0.05		P<0.05		P<0.05		P<0.05		P<0.05		P<0.05	
F-value	4980354.164		6814195.355		5914163.976		398792.0103		6213773.44		3607422.565	

The percentage of individuals linked to HIV care remained consistently above 80% throughout the observed years, ranging from 80.2% in 2018 to 82.4% in 2020, with a slight dip to 81.6% in 2022. The highest recorded number of cases was in 2022 (29,753 cases), reflecting continued efforts to connect individuals to care services.

PrEP coverage significantly improved from 18.2% in 2018 (221,026 cases) to 36% in 2022 (437,425 cases). The most substantial increase occurred between 2020 (24.8%) and 2022 (36%), highlighting the growing adoption of preventive measures. The COVID-19 pandemic in 2020 did not appear to significantly hinder PrEP accessibility, though the rate of increase accelerated post-pandemic. The percentage of individuals receiving HIV medical care remained relatively stable, fluctuating between 74.1% in 2020 and 76% in 2019. The highest recorded number was in 2022 (769,575 cases, 75.6%), demonstrating ongoing efforts to ensure continuity of care for people living with HIV. The rate of unstable housing or homelessness among individuals with HIV showed a gradual decline from 21.0% in 2018 to 17.0% in 2021, with a slight increase to 17.9% in 2022. The highest rate was recorded in 2018 (21.0%), while the lowest was in 2021 (17.0%), indicating improvements in housing support services over time. The p-value for all healthcare access and social factors variables indicated statistically significant (p < 0.05) differences over the years.

The percentage of individuals aware of their HIV status increased steadily from 85.9% in 2018 to 87.2% in 2022. The lowest recorded percentage was in 2018 (1,003,086 cases, 85.9%), while the highest was in 2022 (1,079,751 cases, 87.2%). Notably, during the COVID-19 pandemic in 2020, knowledge of status remained relatively stable at 86.5%, indicating resilience in diagnostic efforts despite healthcare disruptions. Further, the percentage of individuals linked to HIV care remained consistently above 80% throughout the observed years, ranging from 80.2% in 2018 to 82.4% in 2020, with a slight dip to 81.6% in 2022. The highest recorded number of cases was in 2022 (29,753 cases), reflecting continued efforts to connect individuals to care services.

Pre-exposure prophylaxis (PrEP) coverage significantly improved from 18.2% in 2018 (221,026 cases) to 36% in 2022 (437,425 cases). The most substantial increase occurred between 2020 (24.8%) and 2022 (36%), highlighting the growing adoption of preventive measures. The COVID-19 pandemic in 2020 did not appear to significantly hinder PrEP accessibility, though the rate of increase accelerated post-pandemic. The percentage of individuals receiving HIV medical care remained relatively stable, fluctuating between 74.1% in 2020 and 76% in 2019. The highest recorded number was in 2022 (769,575 cases, 75.6%), demonstrating ongoing efforts to ensure continuity of care for people living with HIV. The rate of unstable housing or homelessness among individuals with HIV showed a gradual decline from 21.0% in 2018 to 17.0% in 2021, with a slight increase to 17.9% in 2022. The highest rate was recorded in 2018 (21.0%), while the lowest was in 2021 (17.0%), indicating improvements in housing support services over time. The p-value for all healthcare access and social factors variables indicated statistically significant (p < 0.05) differences over the years.

## Discussion

Social determinants of health, including stigma, housing stability, and access to healthcare, play a critical role in shaping HIV-related outcomes. Our analysis indicates that HIV stigma, although gradually decreasing, remains a significant barrier to care (UNAIDS, 2021) [11]. The Earnshaw et al., 2019 study has shown that stigma negatively impacts treatment adherence and engagement in care, often leading to poorer health outcomes [12]. The persistence of unstable housing and homelessness among people with HIV further exacerbates disparities in healthcare access, as supported by research by Aidala et al. (2020), highlighting the link between housing insecurity and lower retention in HIV care [13]. The study examined HIV stigma's impact on physical and mental health in people living with HIV (PLHIV). Minority groups reported higher stigma levels, which correlated with poorer health outcomes, emphasizing the need for targeted stigma reduction interventions [14]. Addressing these social determinants through policy interventions and targeted programs is essential for improving health equity.

The increasing HIV prevalence observed in this study is consistent with findings from global epidemiological trends. The decline in new HIV infections suggests that prevention strategies such as pre-exposure prophylaxis (PrEP) and expanded HIV testing programs are effective [15]. However, the temporary decline in diagnoses during the COVID-19 pandemic in 2020 indicates potential disruptions in healthcare access, which aligns with studies by Moitra, Ethan et al. showing reduced HIV screening and delayed care during public health crises [16]. The resurgence of diagnoses in 2022 suggests a rebound effect as healthcare systems adapted to pandemic challenges [17].

Our findings highlight a stable yet slightly fluctuating rate of linkage to HIV care, with over 80% of diagnosed individuals engaged in treatment. This aligns with previous research (Higa et al. 2016) demonstrating that early linkage to care is associated with better viral suppression outcomes [18]. The high percentage of individuals receiving HIV medical care throughout the study period indicates that healthcare systems have largely succeeded in maintaining continuity of care despite external disruptions. However, some studies suggest that marginalized populations, including racial and ethnic minorities, still face barriers in accessing timely care. Addressing these disparities through culturally competent care models and targeted outreach programs remains a priority.

HIV viral suppression rates in this study showed a steady increase from 2018 to 2022, reinforcing the effectiveness of antiretroviral therapy (ART) adherence strategies. Previous studies (Byrd et al., 2020) indicate that adherence to ART is crucial for achieving viral suppression and preventing HIV transmission [19]. The stable rates of medical care receipt and viral suppression suggest that adherence support programs, such as medication adherence counseling and community-based interventions, are having a positive impact [20]. However, barriers such as mental health issues and substance use continue to affect adherence rates in some populations. Integrated healthcare approaches that address these co-occurring conditions could further improve treatment outcomes [21].

The substantial increase in PrEP coverage from 2018 to 2022 demonstrates progress in HIV prevention efforts. Studies have shown that PrEP is highly effective in reducing the risk of HIV acquisition, particularly among high-risk populations [22]. However, disparities in PrEP uptake remain a challenge, with lower usage among racial minorities and economically disadvantaged groups [23]. Strategies to improve PrEP access include expanding provider education, reducing cost barriers, and implementing community outreach programs [24]. The increase in PrEP prescriptions following the COVID-19 pandemic suggests a renewed focus on prevention efforts as healthcare services recovered.

Policy interventions play a crucial role in shaping HIV outcomes. The Affordable Care Act and Medicaid expansion have been linked to increased access to HIV care and PrEP services [25]. Structural interventions, such as harm reduction programs and housing assistance initiatives, have also been shown to improve engagement in HIV care. Our findings underscore the importance of sustaining these programs to ensure continued progress in the fight against HIV. Future policies should prioritize addressing social determinants of health to achieve health equity for all individuals at risk for or living with HIV [26].

This study highlights key areas for future research, including the long-term impact of COVID-19 on HIV care, the effectiveness of new prevention technologies, and strategies to reduce disparities in HIV outcomes. Public health initiatives should focus on expanding access to comprehensive HIV care, reducing stigma, and addressing socioeconomic barriers to treatment and prevention. Strengthening collaborations between healthcare providers, community organizations, and policymakers will be essential in achieving global HIV targets [27].

Strengths and limitations of this study

This study has several strengths, including the use of comprehensive national surveillance data from the NCHHSTP database, ensuring a robust analysis of HIV trends in the U.S. Covering five years, it captures longitudinal patterns and the impact of external factors like COVID-19. The inclusion of multiple indicators, from mortality rates to healthcare access, provides a broad understanding of the epidemic.

Nevertheless, reliance on passive surveillance introduces reporting biases, particularly in rural and marginalized communities, where case underreporting may lead us to underestimate new diagnoses and mortality rates. To address this, future research should integrate active case‐finding approaches (e.g., capture-recapture) and link NCHHSTP records with electronic health records or Medicaid/Medicare claims to validate and enrich case ascertainment. The coarse categorization of socioeconomic and stigma variables further limits our ability to detect within‑group heterogeneity; subsequent studies would benefit from geocoded census‑tract indices, detailed stigma scales, and multilevel modeling to disentangle individual versus neighborhood effects. While we attribute temporal shifts largely to the pandemic, we did not adjust for contemporaneous policy changes, such as telehealth expansion or Medicaid modifications, that may have independently shaped care access; future analyses should employ interrupted time‐series or difference‑in‑differences designs incorporating state‑level policy covariates to isolate causal impacts. Finally, the absence of qualitative data constrains our insight into patient experiences and barriers; embedding in‑depth interviews or focus groups with underrepresented PLHIV populations in mixed‐methods frameworks will be essential to contextualize and deepen the quantitative findings.

## Conclusions

The analysis of HIV trends highlights progress in healthcare access, HIV epidemiology, and mortality rates. AIDS-related and HIV-related deaths rose during the COVID-19 pandemic, peaking in 2021 before declining in 2022. Despite this, HIV viral suppression improved, reflecting better treatment adherence. The decline in new infections and rising prevalence indicate enhanced diagnosis and treatment efforts. AIDS classifications fell from 2018 to 2020 but rebounded in 2022, likely due to pandemic-related healthcare disruptions. The sharp drop in HIV diagnoses in 2020 underscores these challenges. Positive trends include greater awareness of HIV status, improved PrEP coverage, and stable linkage to care. However, persistent issues like HIV stigma and unstable housing require continued investment in prevention, treatment, and social support.

To translate these findings into sustained epidemic control, we propose several policy advancements. First, federal and state health agencies should mandate the integration of HIV services into emergency preparedness frameworks, ensuring that testing, treatment, and PrEP delivery are classified as essential during crises. Second, expand Medicaid and Ryan White Program funding to include housing vouchers and supportive services for unstably housed individuals with HIV, thereby addressing a key social determinant. Third, incentivize stigma‑reduction initiatives in healthcare settings by tying performance metrics, such as patient‑reported stigma scores and viral suppression gaps, to provider reimbursement. Finally, support telehealth and mobile outreach programs in rural and underserved areas through dedicated grant programs, reducing geographic and transportation barriers to care.
